# Porcine epidemic diarrhea virus causes diarrhea by activating EGFR to regulates NHE3 activity and mobility on plasma membrane

**DOI:** 10.3389/fmicb.2023.1237913

**Published:** 2023-11-03

**Authors:** YiLing Zhang, Shujuan Zhang, Zhiwei Sun, Xiangyang Liu, Guisong Liao, Zheng Niu, ZiFei Kan, ShaSha Xu, JingYi Zhang, Hong Zou, Xingcui Zhang, ZhenHui Song

**Affiliations:** ^1^School of Animal Medicine, Southwest University Rongchang Campus, Chongqing, China; ^2^Department of Animal Science and Technology, Three Gorges Vocational College, Chongqing, China; ^3^Department of Preventive Veterinary Medicine, College of Animal Medicine, Xinjiang Agricultural University, Xinjiang, China; ^4^College of Veterinary Medicine, Northwest Agriculture and Forestry University, Shanxi, China; ^5^School of Medicine, University of Electronic Science and Technology, Chengdu, China; ^6^Immunology Research Center, Institute of Medical Research, Southwest University, Chongqing, China

**Keywords:** PEDV, sodium-hydrogen exchanger NHE3, EGFR, diarrhea, piglets

## Abstract

As part of the genus Enteropathogenic Coronaviruses, Porcine Epidemic Diarrhea Virus (PEDV) is an important cause of early diarrhea and death in piglets, and one of the most difficult swine diseases to prevent and control in the pig industry. Previously, we found that PEDV can block Na^+^ absorption and induce diarrhea in piglets by inhibiting the activity of the sodium-hydrogen ion transporter NHE3 in pig intestinal epithelial cells, but the mechanism needs to be further explored. The epidermal growth factor receptor (EGFR) has been proved to be one of the co-receptors involved in many viral infections and a key protein involved in the regulation of NHE3 activity in response to various pathological stimuli. Based on this, our study used porcine intestinal epithelial cells (IPEC-J2) as an infection model to investigate the role of EGFR in regulating NHE3 activity after PEDV infection. The results showed that EGFR mediated viral invasion by interacting with PEDV S1, and activated EGFR regulated the downstream EGFR/ERK signaling pathway, resulting in decreased expression of NHE3 and reduced NHE3 mobility at the plasma membrane, which ultimately led to decreased NHE3 activity. The low level of NHE3 expression in intestinal epithelial cells may be a key factor leading to PEDV-induced diarrhea in newborn piglets. This study reveals the importance of EGFR in the regulation of NHE3 activity by PEDV and provides new targets and clues for the prevention and treatment of PEDV-induced diarrhea in piglets.

## 1. Introduction

PEDV is one of the main pathogens that cause diarrhea in piglets, it is a gastrointestinal infection with high mortality, manifested by jet diarrhea, rapid dehydration and severe vomiting (No et al., 2015; Chen et al., 2019; Niu et al., 2021). Indeed, 80–100% of piglets die within a few days after infection (Alvarez et al., 2015; Lee, 2015). In recent years, because of the continuous emergence of new epidemic strains of PEDV, traditional vaccination has not achieved the expected prevention and control effect, and large-scale outbreaks continue to occur, bringing serious economic losses to the pig industry (Wang et al., 2020). The target organ of PEDV infection in the host is the pig’s small intestine, which can cause intestinal cell dysfunction and abnormal expression of channel proteins related to water and salt metabolism, resulting in disruption of the balance between absorption and secretion of intestinal substances and loss of water and electrolytes, ultimately leading to vomiting, reduced appetite, and impaired absorption diarrhea (Song et al., 2015). Therefore, determining the etiology and pathogenic mechanism of PEDV-induced diarrhea could provide a more effective method to its prevention and control.

Severe dehydration due to acute diarrhea is the key factor leading to death in newborn piglets. Diarrhea is associated with impaired Na^+^ absorption by IPEC-J2 cells (Field, 2003); but the mechanism of sodium imbalance diarrhea due to PEDV needs to be further investigated. Na^+^/H^+^ exchanger 3 is one of the transmembrane transporter proteins that are essential for mediating Na^+^/H^+^ homeostasis in the gut, playing a major role in regulating cellular pH and the homeostasis of the intestinal microenvironment (No et al., 2015). Changes in its activity and the amount of expression are closely related to the onset of diarrhea. When the activity of NHE3 is inhibited for a long time, it can lead to the occurrence of disease, and even death in severe cases (Anbazhagan et al., 2018). NHE3 is transported in the plasma membrane and the intracellular circulation of small intestinal villous epithelial cells, and the sodium-hydrogen exchange activity is mainly influenced by the amount of NHE3 transferred to the plasma membrane. Therefore, regulation of the functional activity of NHE3 is closely related to its mobility at the plasma membrane.

Signaling pathways activated by the epidermal growth factor receptor (EGFR) play an important role in cell proliferation, apoptosis and viral infection. EGFR is the transmembrane receptor for most viruses, including TGEV, which helps the virus invade the host and activates related signaling pathways (Hu et al., 2018). A recent study found that activation of EGFR occurs at an early stage of PEDV infection and might interact directly with viral S proteins to mediate PEDV invasion (Yang L. et al., 2018), the mechanism of action of which is unclear.

Further studies in our laboratory have shown that specific inhibition of NHE3 activity in IPEC-J2 cells leads to watery diarrhea and severe dehydration in piglets (Niu et al., 2021). Furthermore, PEDV infection inhibited the expression of NHE3 in IPEC-J2 cells and reduced extracellular Na^+^ uptake by the cells. Thus, the aim of this study was to explore the role of EGFR in the regulation of NHE3 activity by PEDV infection is an important reference for finding the targets of action against PEDV diarrhea.

## 2. Materials and methods

### 2.1. Cells, viruses, and reagents

IPEC-J2 and African green monkey kidney cells (Vero) were both cultured in DMEM medium containing 10% fetal bovine serum, 1% double antibody in a 37°C, 5% CO_2_ cell culture incubator. The cells mentioned above were all pre-preserved in the laboratory. The PEDV-LJX variant was kindly presented by Guangliang Liu, a researcher from Lanzhou Veterinary Research Institute. Recombinant human EGF from Gibco (Grand Island, NY, USA) at 10 ng/mL. The tyrosinase inhibitor AG1478 was purchased from Apexbio (Houston, TX, USA) and used at a dose of 30 μM.

### 2.2. Total cellular proteins and membrane protein extraction

IPEC-J2 cells were seeded in 60 mm dishes. An experimental group and a control group were set up, and each group was replicated three times independently. When cells grew to 90%, PEDV virus fluid (MOI = 0.1), or an optimal dose of EGFR modulator, was inoculated at 2 h and 72 h, respectively. The proteins were extracted using Beyotime’s cell membrane and plasma protein extraction kits, added with 6 × Loading Buffer (TransGen Biotech, China), denatured at 100°C for 10 min, cooled, and stored at −20°C before use.

### 2.3. Western blotting

Equal amounts of protein samples were separated by SDS-PAGE gel electro-phoresis and transferred to polyvinylidene fluoride membranes. The membranes was blocked with 5% skimmed milk powder diluted by TBST. After incubation overnight at 4°C with the appropriate primary antibody, the membrane was washed three times with 1 × TBST for 10 min/time and then incubated with goat anti-rabbit secondary antibody or goat anti-mouse secondary antibody (Proteintech, Rosemont, IL, USA) for 90 min on a shaker at room temperature. Images of the immunoreactive proteins on the membrane were chromogenic using the FX5 imaging system (VILBER, Marne-la-Vallée cedex 3, France). The grayscale values were analyzed. The phospho-EGFR (Tyr1068) antibody was purchased from Cell Signaling Technology (Danvers, MA, USA). Antibodies recognizing EGFR, SLC9A3 (NHE3), β-Actin, β-Tubulin (Rabbit Antibody), 6 × His (His-Tag), and FLAG® tag were purchased from Proteintech. The phospho-extracellular kinase (ERK)1/2 (Thr202 + Tyr204) antibody was purchased from Beijing BoaoSen Biotechnology Co. (Beijing, China).

### 2.4. Half of the tissue culture infected dose (TCID_50_)

After IPEC-J2 cells had grown to spread all over the cell bottles, pre-treatment of cells with 10 ng/mL of EGF or 30 μM of AG1478 in 2 mL, and then inoculated with 10^5^ TCID_50_/mL PEDV-LJX strain, and both virus-infected and blank control groups were established. After incubation with the virus for 1.5 h, the cells were freeze-thawed three times and then centrifuged at 12000 × g for 10 min. The supernatant was collected into centrifuge tubes, and the TCID_50_ of each group of PEDV was determined in Vero cells according to the Reed-Muench method.

### 2.5. Co-immunoprecipitation (Co-IP)

Sequences of the coding regions of the PEDV S and EGFR genes registered in GenBank according to the NCBI, PEDV S1 (637–2,127 bp) was selected and constructed into vector pTT5 as a Flag tag fusion. The fragment of EGFR encoding the extracellular region (139–1,473 bp) was constructed into vector pTT5 as a His tag fusion. The above gene fragments were synthesized, and the recombinant plasmids were constructed, and sequenced by Wuhan GeneCreate Biological Engineering Co., Ltd. (Wuhan, China). Splicing HEK-293 T cells into 60 mm dishes, and when the cells grew to about 80% confluence, they were transfected with the constructed eukaryotic expression vector plasmids of PEDV S1 and EGFR extracellular region. At 48 h after transfection, samples were prepared for immunoprecipitation and the target bands were detected using western blotting.

### 2.6. Fluorescence recovery after photobleaching (FRAP)

IPEC-J2 cells were inoculated with 1 × 10^4^ cells per well in a confocal dish (medium without antibiotics), and when the cells reached 80% growth, the cells were washed 3 times with PBS. After 6 h of plasmid transfection, the AG1478 treatment solution was slowly added from the edge of the confocal dish, mixed well and incubated at 37°C with 5% CO_2_ for 24 h. The pEGFP-NHE3 DMSO treatment group was treated with the same dose of DMSO for 24 h. The pEGFP-NHE3 EGF treatment group was replaced with serum-free phenol red-free DMEM after 6 h of plasmid transfection. After the above groups had reached the treatment time point, they were washed twice with PBS and replaced with phenol red-free maintenance medium for FRAP assay. A Zeiss LSM 800 laser confocal microscope with a 63 × oil objective (numerical aperture of 1.4) was used to observe and photograph the cell samples, and the ROI was used to observe the real-time fluorescence intensity of the three areas. Finally, the ZEN (blue) software was used to export the data and to compare the fluorescence intensity of the bleached areas throughout the process. When the test is completed for all groups, use ZEN (blue) software to export the data and analyze the fluorescence intensity of the bleached area throughout the process (Yang Z. et al., 2018).

### 2.7. Animal experiments

Nine 3-day-old lactating Rongchang piglets were randomly divided into Control, PEDV-infected and Tenapanor groups, with three piglets in each group. 10 mL of saline was administered to the Mock group, 10 mL of 1 × 10^6^ TCID_50_/mL of PEDV LJX strain was administered orally to the PEDV-infected group, and 10 mL of Tenapanor was administered to the Tenapanor group. After inoculation, clinical signs, such as diarrhea, were assessed daily. After significant diarrhea developed in the PEDV group, piglets from both groups were uniformly dissected to observe intestinal lesions.

All animal experiments were approved by the Southwestern University Institutional Animal Care and Use Committee (animal protocol approval number: CQLA-2021-0122). The National Institutes of Health guidelines for the performance of animal experiments were followed.

### 2.8. Data analysis

All statistical analyses were performed using GraphPad Prism 8.0 (GraphPad Inc., La Jolla, CA, USA). All data are expressed as the mean ± SD or standard error of the mean (SEM) of three separate repetitions. ANOVA and *t*-tests were used to analyze significant differences in *p* values (*p* * < 0.05; *p* ** < 0.01; *p* *** < 0.001).

## 3. Results

### 3.1. NHE3 inhibitor induces diarrhea in piglets

**Figure 1:**
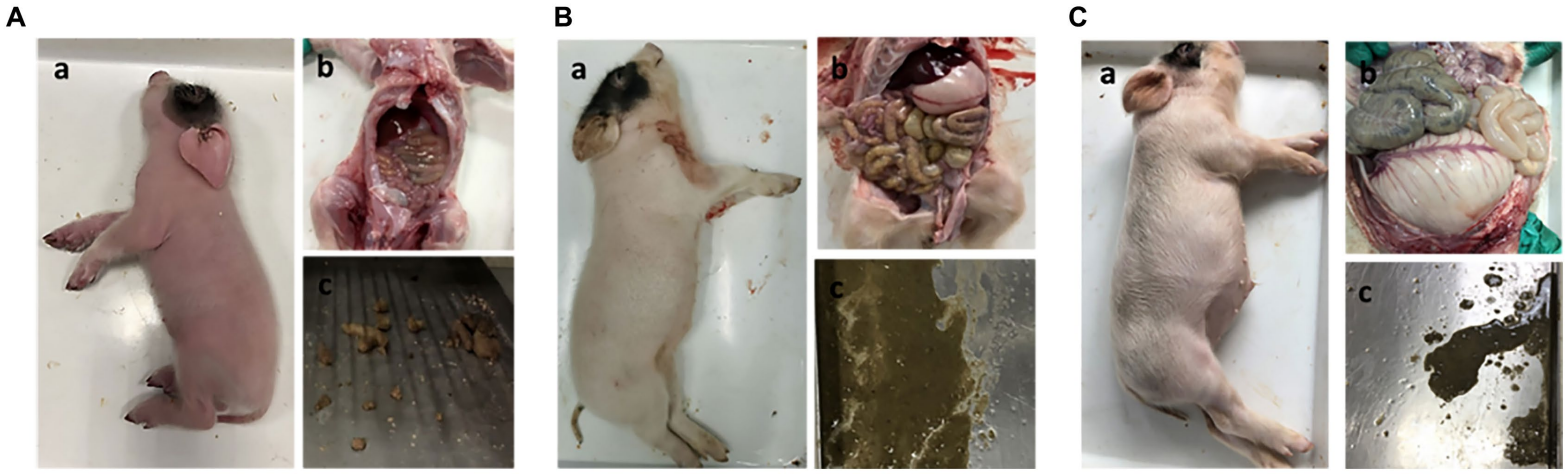
Clinical autopsy diagram of piglets with diarrhea. **(A)** Anatomy of a normal piglet; **(B)** Anatomy of a PEDV-infected piglet; **(C)** Anatomy of a NHE3 inhibitor-fed piglet.

To determine whether NHE3 is associated with piglet diarrhea, piglets in the control group received 10 mL of saline orally, PEDV-infected groups were orally infected with the PEDV-LJX strain, and the NHE3 inhibitor-fed piglets received Tenapanor at a concentration of 15 mg/kg. The anatomical results showed that control piglets had normal, dry, and formed stools (Figure 1A). However, the PEDV-infected piglets and the NHE3 inhibitor-fed piglets showed dilute watery diarrhea and their gastrointestinal tracts were thinning with transparency, and swollen (Figures 1B,C). These studies demonstrate that reducing the activity of the transporter protein NHE3 in piglet small intestinal epithelial cells leads to severe diarrhea and rapid dehydration in piglets.

### 3.2. The protein expression level of NHE3 protein was dose-dependent with PEDV titers

**Figure 2:**
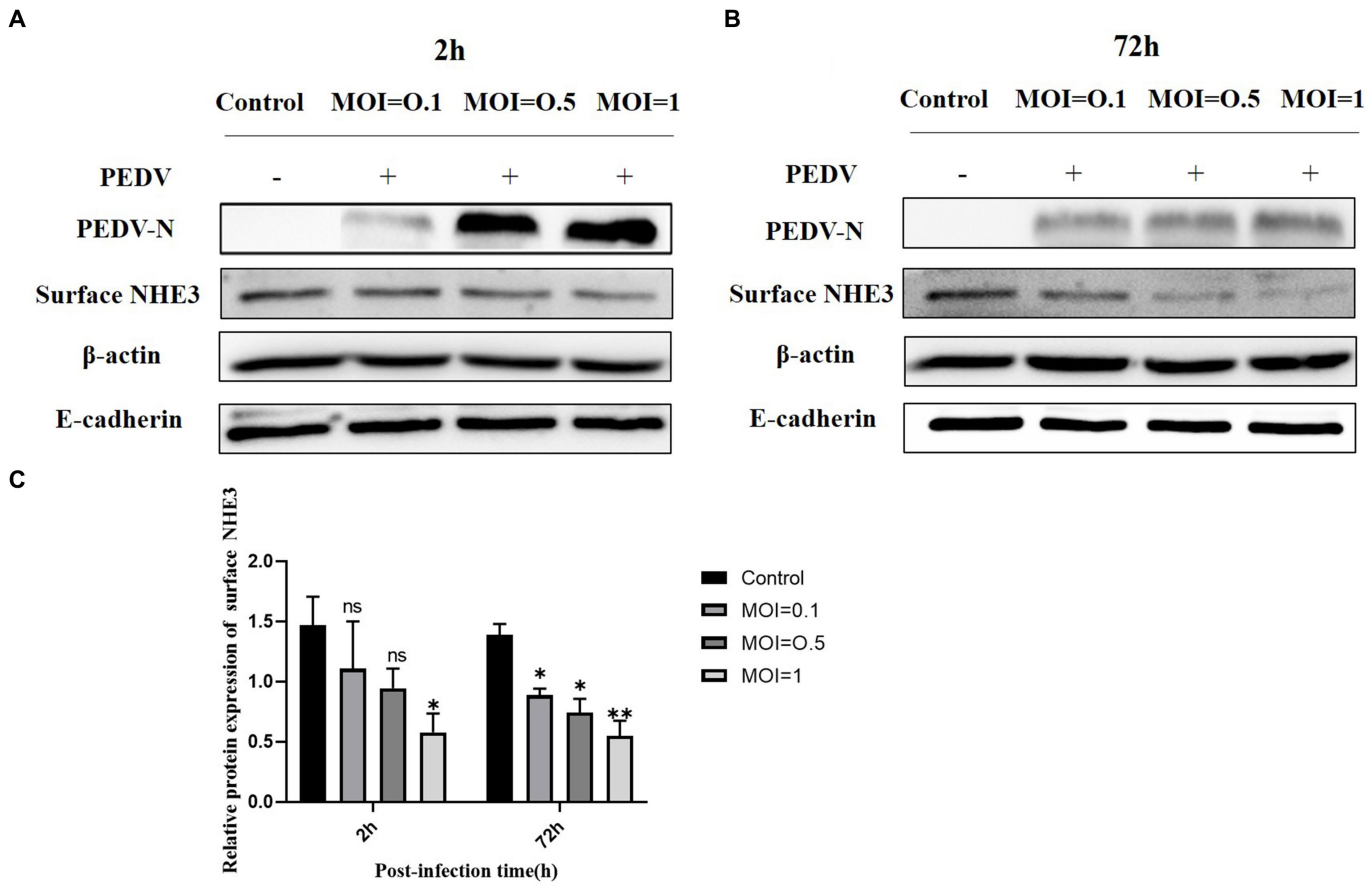
NHE3 protein levels at different titres of PEDV infection. **(A)** Western blotting results for surface NHE3 at 2 h after PEDV infection; **(B)** Western blotting results for surface NHE3 at 72 h after PEDV infection; **(C)** Grayscale analysis results for surface NHE3 protein at 2 and 72 h post PEDV infection.

To further investigate the relationship between PEDV and NHE3, we detected the NHE3 protein expression levels at different titers of PEDV by Western blot. The results showed that when PEDV was infected at MOI = 1.0 or 0.5 for 2 h, the level of NHE3 decreased significantly at MOI = 0.1 (Figures 2A,C). 72 h after PEDV infection, the level of NHE3 decreased significantly at MOI = 0.1, 0.5 and 1 (Figures 2B,C). These results indicated that NHE3 levels decreased in a dose-dependent manner according to PEDV titers. The higher of PEDV titer, the more pronounced of the decrease in NHE3 levels.

### 3.3. PEDV infection induces EGFR phosphorylation on IPEC-J2 cells

**Figure 3:**
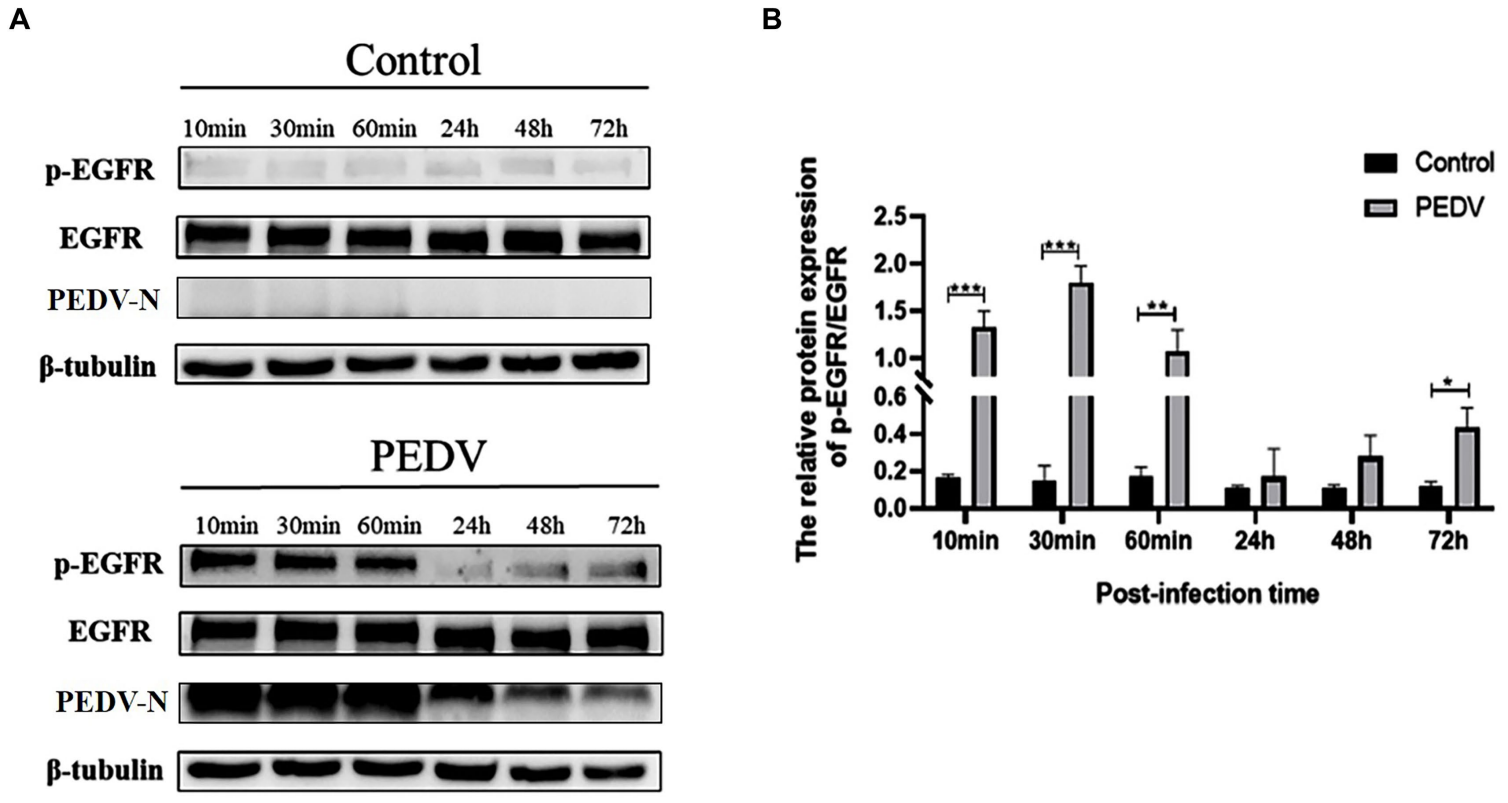
Levels of p-EGFR/EGFR at different points of time after PEDV infection of IPEC-J2 cells. **(A)** Graph of p-EGFR/EGFR at different points of time after PEDV infection with IPEC-J2 cells according to the western blotting results; **(B)** Grayscale analysis of p-EGFR/EGFR levels.

To verify that PEDV infection activates EGFR, activation of the EGFR was evaluated by detecting the level of phosphorylated EGFR (p-EGFR) at different time points after PEDV infection of IPEC-J2 cells. The Western-blot results (Figure 3) indicated that the level of p-EGFR increased significantly during the pre-PEDV infection stage (10, 30, and 60 min) compared with the control group. In the advanced stages of PEDV infection (24, 48 h), p-EGFR levels remained upregulated but not significantly, then significantly higher at 72 h (0.01 < *p* < 0.05). These findings showed that PEDV infection rapidly activates EGFR, contributing to its phosphorylation and increased its expression.

### 3.4. PEDV interacts with the EGFR extracellular region through the S1 protein

**Figure 4:**
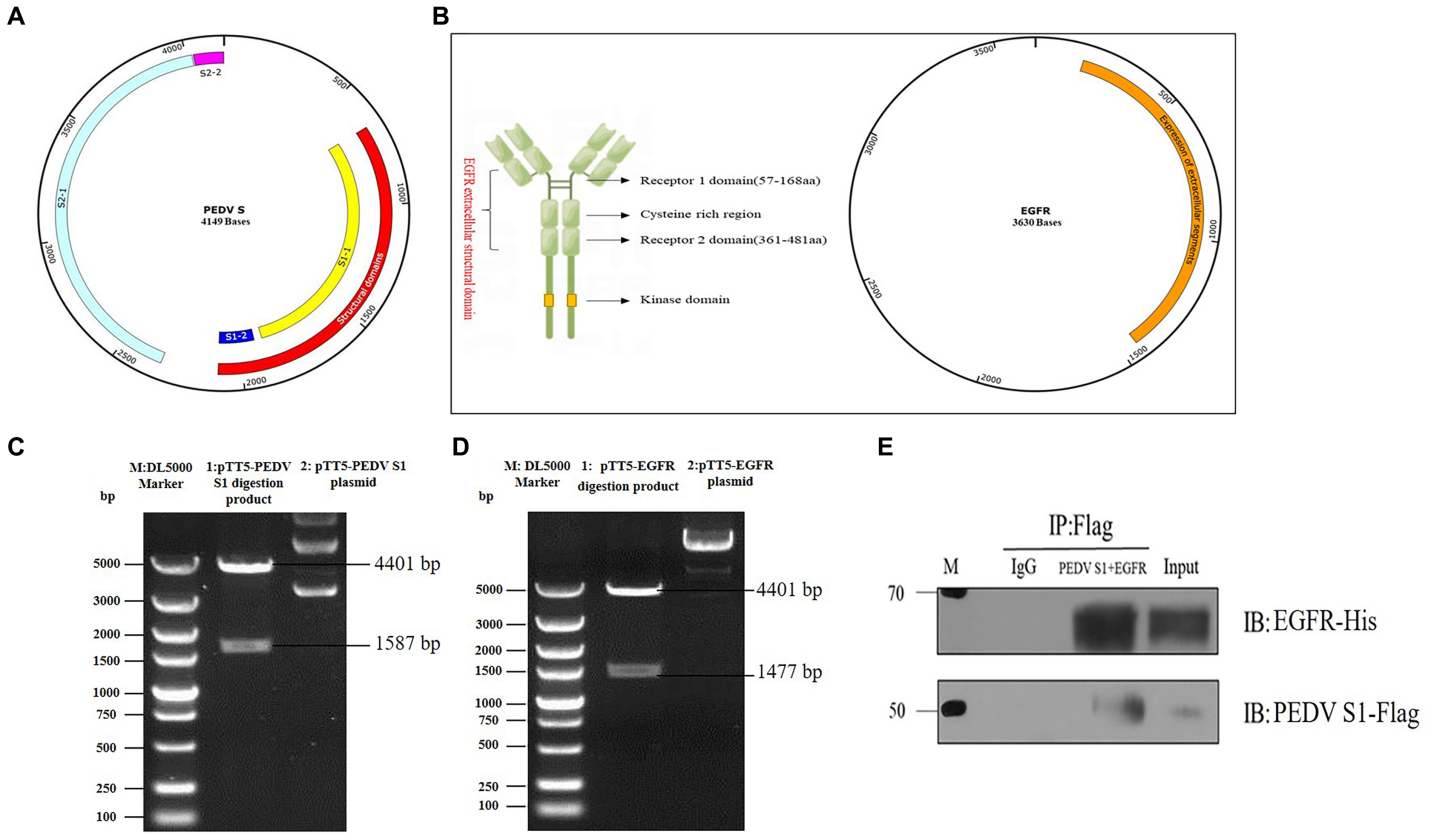
Vector construction of PEDV S1 and EGFR. **(A)** Construction of PEDV S1 eukaryotic expression vector (red); **(B)** Construction of EGFR extracellular structural domain and eukaryotic expression vector (orange); **(C)** Double digestion identification of the PEDV S1 eukaryotic expression vector (M: DL5000 Marker; 1: pTT5-PEDV S1 digestion product; 2: pTT5-PEDV S1 plasmid); **(D)** Double digestion identification of the EGFR eukaryotic expression vector (M:DL5000 Marker; 1: pTT5-EGFR digestion product; 2: pTT5-EGFR plasmid); **(E)** Co-IP of PEDV S1 and the EGFR extracellular region.

The S protein of PEDV is a type I transmembrane glycoprotein consisting of two structural domains, S1 and S2, responsible for binding and fusion with the host cell. EGFR exists as a dimer on the cell membrane surface and the functional region is divided into an extracellular receptor binding region, a transmembrane region and an intracellular kinase structural domain (Figure 4B). To investigate the interaction between the S protein and EGFR, the PEDV S gene (637 bp-2127 bp) (Figure 4A) and the EGFR gene (139-1473 bp) were ligated onto the ptt5 vector (Figure 4B) with different tags (flag and his) to identify the size by PCR through Wuhan GeneCreat (Figures 4C,D). Immunoprecipitation was used to test whether the PEDV S1 structural domain could directly interact with the extracellular region of EGFR. Lysates from HEK 293 T cells co-transfected with pTT5-PEDV S1 and pTT5-EGFR plasmids were subjected to western-blot after Co-IP to detect the results. The results confirmed the interaction of the PEDV S1 structural domain with the extracellular region of EGFR (Figure 4E), indicating that EGFR mediates PEDV invasion through binding of the extracellular receptor binding region to the PEDV S1 structural domain.

### 3.5. EGFR is involved in PEDV invasion of IPEC-J2 cells

**Figure 5:**
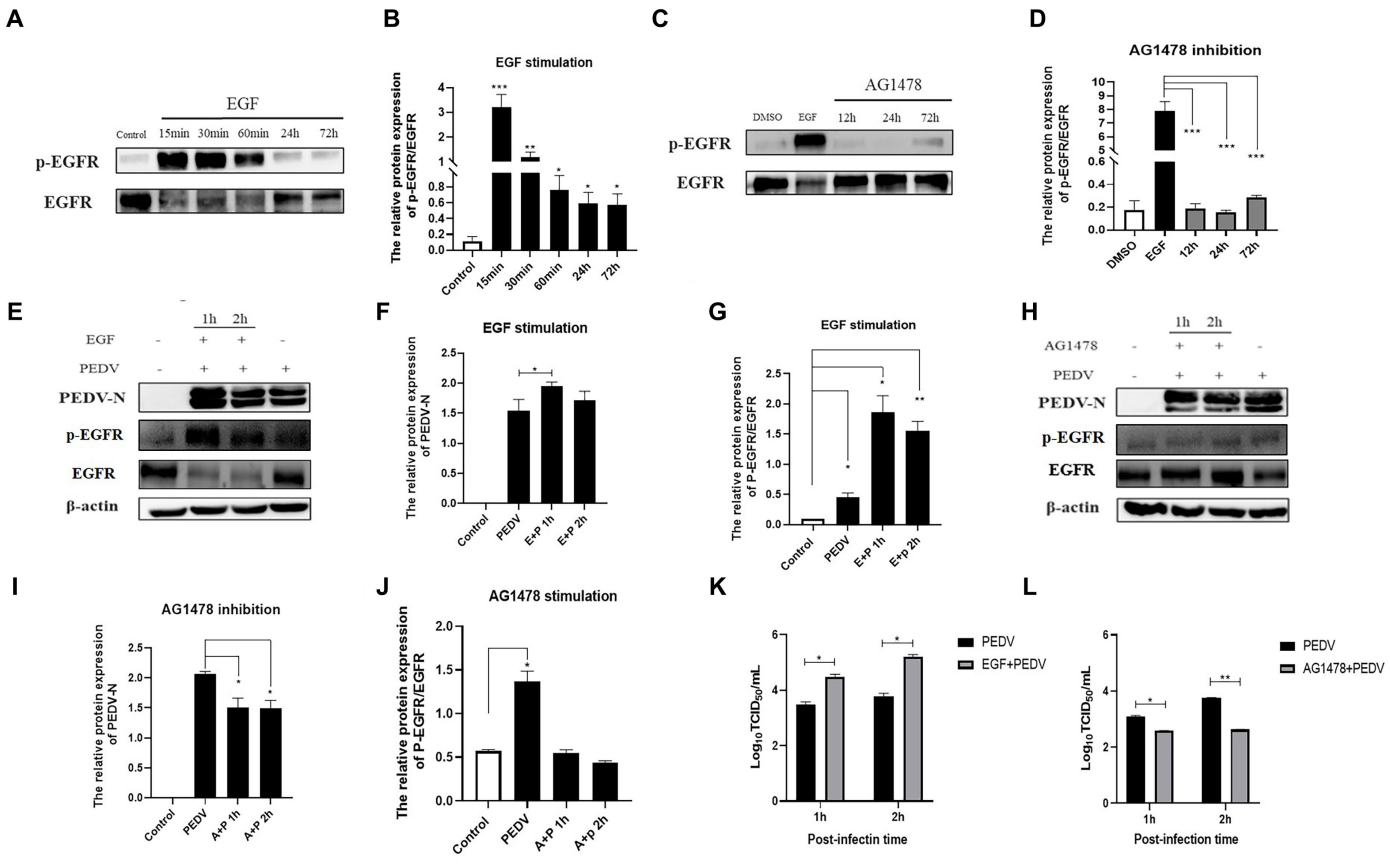
EGFR regulates PEDV invasion into IPEC-J2 cells. **(A)** Western blotting results for p-EGFR/EGFR after EGF treatment; **(B)** Grayscale analysis results for p-EGFR/EGFR after EGF treatment; **(C)** Results of western-blot for p-EGFR/EGFR after AG1478 treatment; **(D)** Grayscale analysis results for p-EGFR/EGFR after AG1478 treatment; **(E)** Results of western-blot for PEDV N, p-EGFR, EGFR protein after EGF treatment; **(F)** Grayscale analysis results of PEDV N protein after EGF treatment; **(G)** Grayscale analysis results for p-EGFR/EGFR after EGF treatment; **(H)** Western blotting results for PEDV N, p-EGFR, EGFR protein after AG1478 treatment; **(I)** Grayscale analysis results for PEDV N protein after AG1478 treatment; **(J)** Grayscale analysis results for p-EGFR/EGFR after AG1478 treatment; **(K)** Virus titer detected by TCID_50_ after EGF treatment; **(L)** Virus titer detected by TCID_50_ after AG1478 treatment.

Based on the fact that PEDV infection activates EGFR, we investigated whether altering the phosphorylation level of EGFR would affect PEDV invasion. In this experiment, EGFR phosphorylation was regulated by EGF (a specific activator of EGFR) and AG1478 (a specific inhibitor of EGFR). It has been shown that EGFR phosphorylation can be induced by treating cells with 10 ng/mL of EGF, and previous studies in our laboratory have shown that 30 μM of AG1478 has the best inhibitory effect on EGFR phosphorylation and it is not toxic to IPEC-J2 cells (Yang Z. et al., 2018). The effect of EGFR modulators on phosphorylated EGFR levels was examined using western blotting, which showed that the optimal duration of action was 15 min for EGF (Figures 5A,B) and 24 h for AG1478 (Figures 5C,D). IPEC-J2 cells were pretreated with the optimal dose and duration of the EGFR modulators determined above, the expression of PEDV N protein was detected by western blotting and the viral titer was determined by TCID_50_ after 1 h and 2 h of PEDV incubation. The results indicated that PEDV infection increased and EGFR phosphorylation levels were elevated after EGF pretreatment (Figures 5E–G,K), and was significantly higher at 1 h of PEDV infection (0.01 < *p* < 0.05), indicating that activation of EGFR promotes PEDV infection. In contrast, PEDV infection as well as EGFR phosphorylation levels was significantly reduced after 24 h of AG1478 pretreatment (0.01 < *p* < 0.05), indicating that inhibition of EGFR activity reduced PEDV infection (Figures 5H–J,L).

### 3.6. Regulation of EGFR activity affects the level of NHE3 protein

**Figure 6:**
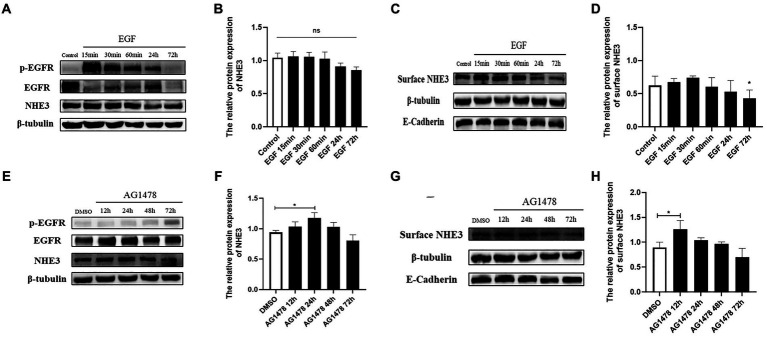
Levels of NHE3 at different time points after regulation of EGFR activity. **(A)** Western blotting results for total NHE3 after EGF treatment; **(B)** Grayscale analysis results for total NHE3 after EGF treatment; **(C)** Western blotting results for membrane-bound NHE3 after EGF treatment; **(D)** Grayscale analysis results for membrane-bound NHE3 after EGF treatment; **(E)** Western blotting results for total NHE3 after AG1478 treatment; **(F)** Grayscale analysis results for total NHE3 after AG1478 treatment; **(G)** Western blotting results for membrane-bound NHE after AG1478 treatment; **(H)** Grayscale analysis results for membrane-bound NHE3 after AG1478 treatment.

To investigate whether there is a direct correlation between EGFR and changes in NHE3, the level of NHE3 was detected after modulating EGFR activity. Western blotting showed that total NHE3 protein levels remained essentially unchanged for the first 60 min after EGFR activation using EGF, and surface NHE3 levels were slightly upregulated in the first 30 min, but decreased at both 24 h and 72 h, in which the decrease at 72 h was significant (Figures 6A–D). Total NHE3 levels and surface NHE3 levels were upregulated for 48 h after EGFR inhibition using AG1478, but were slightly downregulated at 72 h (Figures 6E–H). The above results proved that there is a correlation between EGFR activity and NHE3 levels, and when EGFR activity is inhibited, NHE3 protein levels are upregulated.

### 3.7. PEDV infection regulates NHE3 levels through the EGFR/ERK signaling pathway

**Figure 7:**
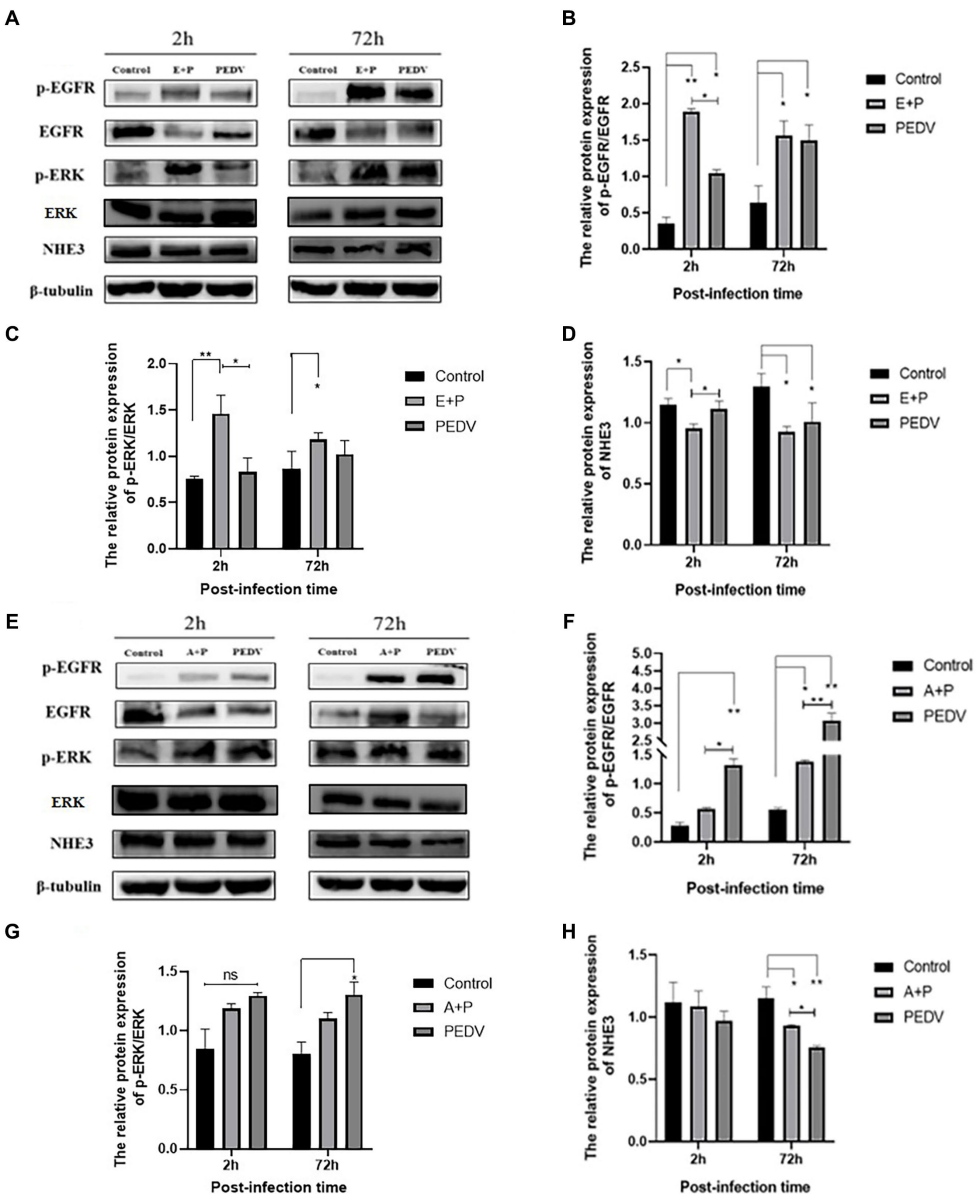
Level of EGFR, ERK, and NHE3 proteins at different times of PEDV infection after regulation of EGFR. **(A)** Western blotting results for p-EGFR, EGFR, p-ERK, ERK, and NHE3 after activation of EGFR; **(B)** Grayscale analysis results for p-EGFR/EGFR; **(C)** Grayscale analysis results for p-ERK/ERK; **(D)** Grayscale analysis results for NHE3; **(E)** Western blotting results of p-EGFR, EGFR, p-ERK, ERK, and NHE3 after inhibition of EGFR; **(F)** Grayscale analysis results for p-EGFR/EGFR; **(G)** Grayscale analysis results for p-ERK/ERK; **(H)** Grayscale analysis results for the NHE3.

To investigate whether PEDV can regulate the level of NHE3 through the EGFR/ERK signaling pathways, PEDV was used to infect IPEC-J2 cells after activation of EGFR, and the levels of key signaling factors in the EGFR/ERK signaling pathway, and the level of NHE3 was examined. The results showed that EGFR was activated after PEDV infected cells for 2 h and 72 h, there was a significant increase in the level of p-EGFR (Figures 7A,B), and the change in level of p-ERK was basically consistent with that of p-EGFR (Figures 7A,C). However, the NHE3 level was downregulated at both 2 h and 72 h of PEDV infection after activation of EGFR activity (0.01 < *p* < 0.05), and the level was lower than that of the PEDV infection alone group (Figures 7A,D). PEDV infection at 2 h and 72 h after inhibition of EGFR activity continued to elevate p-EGFR levels. However, the elevated levels of p-EGFR were significantly higher in the PEDV-infected group than in the group with inhibition of EGFR activity (Figures 7E,F). Changes in the levels of p-ERK were largely consistent with those of p-EGFR and were also elevated (Figures 7E,G). NHE3 was downregulated after PEDV infection at both 2 h and 72 h compared with that of control group after inhibition of EGFR activity (Figures 7E,H), which was significant at 72 h (0.01 < *p* < 0.05). However, its level of downregulation was less significant than that in the PEDV-infected group, with a significant difference at 72 h (0.01 < *p* < 0.05). The above results suggest that PEDV infection leads to notable upregulation of p-EGFR and p-ERK following activation of EGFR activity in IPEC-J2 cells, and that ERK is regulated by EGFR following PEDV infection. This demonstrates that the effect of PEDV infection on NHE3 is regulated by the EGFR/ERK signaling pathway. Activation of EGFR downregulated NHE3 levels more significantly, while inhibition of EGFR activity somewhat attenuated the downregulation of NHE3 in IPEC-J2 induced by PEDV infection.

### 3.8. PEDV infection regulates the mobility of plasma membrane NHE3 through EGFR

**Figure 8:**
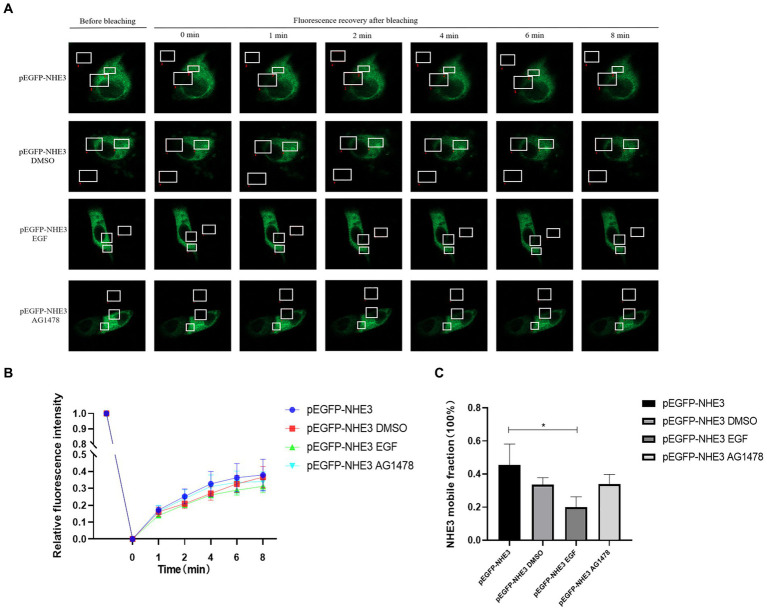
Rate of recovery of NHE3 fluorescence and dynamics at the plasma membrane following modulation of EGFR. **(A)** Dynamics of fluorescent molecules in the bleaching region during FRAP (63×/1.4NA); **(B)** Fluorescence bleaching recovery of NHE3 on cell membranes; **(C)** Dynamic analysis of NHE3 on membranes.

**Figure 9:**
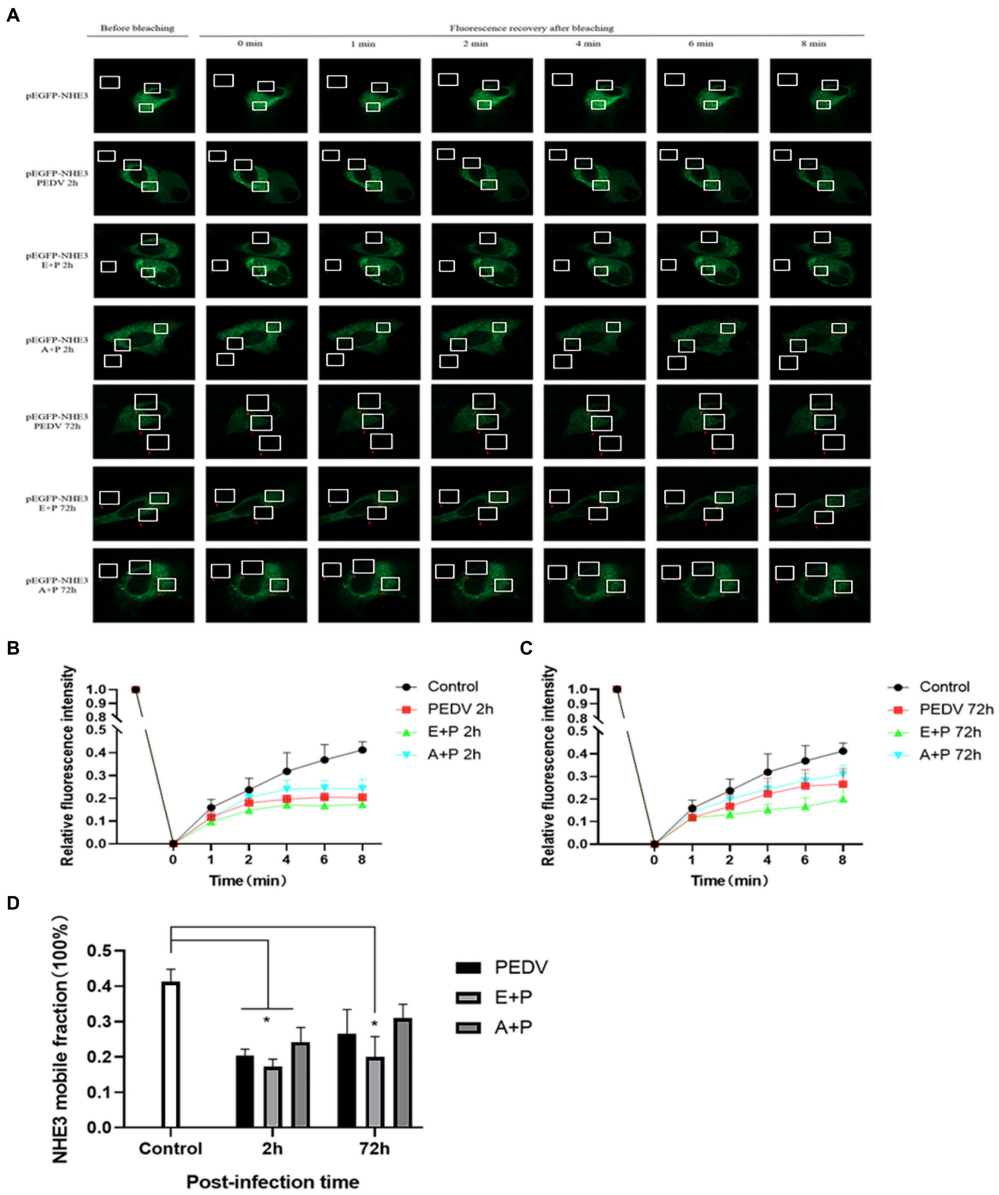
PEDV infection regulates the mobility of plasma surface NHE3 through EGFR. **(A)** Dynamics of fluorescent molecules in the bleaching region during FRAP (63×/1.4NA); **(B)** Fluorescence recovery rate of NHE3 on the cell membrane 2 h after PEDV invasion; **(C)** Fluorescence recovery rate of NHE3 on the cell membrane 72 h after PEDV invasion; **(D)** Analysis of the dynamic fraction of NHE3 on the cell membrane after PEDV invasion.

To better observe the dynamic changes in NHE3 induced by PEDV processing, the impacts of modulating EGFR activity on the mobility of NHE3 across the plasma membrane of IPEC-J2 cells after PEDV infection was examined using FRAP. The fluorescence intensity of the bleached areas in all groups decreased significantly after bleaching and started to recover again with time, indicating that the NHE3 fluorescent molecules remained mobile after PEDV infection (Figures 8A, 9A). The pEGFP-NHE3 group (Control) showed stronger recovery of fluorescence intensity than all PEDV-infected groups, while the pEGFP-NHE3 EGF + PEDV group had the weakest recovery of bleached areas post-bleaching. The fluorescence intensity of the anchored bleached area before and during recovery was analyzed for each group using ZEN (blue) software, which was used to detect the fluorescence recovery rates and dynamic fraction (Mobile fraction, Mf) of each group.

The results showed that the NHE3 fluorescence recovery rate was significantly lower in the EGF-treated group compared with that in the pEGFP-NHE3 group. The fluorescence recovery rate in the AG1478-treated group was higher than that in the DMSO group in the first 6 min and lower than that in the DMSO group after 6 min (Figures 8A,B). However, the NHE3 fluorescence recovery rate in all three groups was lower than the pEGFP-NHE3 group (Figure 8C). This indicated that NHE3 fluorescent bleaching recovery rate was relatively reduced after activation of EGFR, and inhibition of EGFR activity upregulated the fluorescence recovery rate of NHE3. Compared with that in the control group, NHE3 fluorescent bleaching recovery rate in all PEDV-infected groups was weaker at each time point (Figures 9A–C). After activating EGFR, infection with PEDV induced a more noticeable decline in the NHE3 fluorescence recovery rate at 72 h, while inhibiting EGFR activity followed by infection with PEDV relatively upregulated the fluorescence recovery rate of NHE3, and the effect was better at 2 h (Figure 9D). The above results show that compared with the control group, PEDV infection of IPEC-J2 cells reduced the fluidity of NHE3 on the plasma membrane. When EGFR was activated, PEDV infection further reduced the fluidity of NHE3, whereas infection with PEDV after inhibition of EGFR activity could enhance the fluidity of NHE3, indicating that during PEDV infection, the stronger the activity of EGFR on the IPEC-J2 cell membrane, the weaker the fluidity of NHE3, i.e., negative feedback by EGFR regulates the fluidity of NHE3 on the plasma membrane.

## 4. Discussion

It has been well documented that EGFR can act as one of the co-receptors involved in viral infection. Previous studies in our laboratory have found that EGFR promotes the intracellular proliferation of TGEV (Yang Z. et al., 2018), and it is unknown whether EGFR plays an equivalent role in the process of PEDV infection. Therefore, we first verified that PEDV infection could cause EGFR phosphorylation, and the results showed that EGFR phosphorylation levels increased significantly at 10 min of PEDV infection and peaked at 30 min, indicating that viral infection could rapidly induce intracellular EGFR activation, resulting in enhanced EGFR activity (Figure 3). However, in contrast to the results of the identified studies, we found that EGFR phosphorylation levels were again elevated at later stages of PEDV infection (48 h, 72 h), but not as significantly as at earlier stages. It is possible that this is caused by the massive replication of PEDV after cell invasion and in the subsequent release of new viral particles that infect the surrounding host cells.

Viral invasion is the initial step of viral infection. PEDV invasion into host cells is mediated by S glycoproteins attached to specific host receptors, and S proteins can be subdivided into two subunits, S1, which mediates the binding of viruses to surface-specific receptors on host cells, and S2, which is involved in the fusion process of viral and host cell membranes (Kirchdoerfer et al., 2021). In Hu W’s study, p-APN and EGFR were found to be synergistically involved in TGEV invasion, and EGFR can be activated early in PEDV infection to promote adsorption of virus invasion, suggesting that EGFR is most likely one of the invasion receptors of PEDV (Hu et al., 2018). Therefore, this experiment confirmed the direct interaction between the PEDV S1 structural domain and the EGFR extracellular region by constructing eukaryotic expression vectors for PEDV S1 and EGFR extracellular region and immunoprecipitating them after co-transfection with HEK 293 T cells (Figure 4). It indicates that EGFR activation induced by PEDV infection may be mediated by a direct interaction between the EGFR extracellular receptor binding region and PEDV S1 protein.

Since the invasion process of PEDV occurs early in the infection, it has been shown that viral infection can competitively exploit the endocytosis of EGFR and activate EGFR downstream signaling pathways to counteract the host’s antiviral response (Zheng et al., 2014; Perez Verdaguer et al., 2021). Therefore, this experiment next investigated the relationship between EGFR and PEDV invasion. After modulating EGFR activity by EGFR-specific regulators epidermal growth factor (EGF) and tyrosinase inhibitor (AG1478), we examined the viral titer and N protein expression in PEDV-infected IPEC-J2 cells at 1 h and 2 h. We found that activation of EGFR promoted PEDV infection and inhibition of EGFR activity reduced PEDV infection (Figure 5). This validates our conjecture that EGFR can be involved in PEDV invasion of IPEC-J2 cells and that EGFR activation induced by PEDV infection enhances the ability of PEDV to infiltrate.

Epidermal growth factor (EGF) is the most primitive member of the EGF ligand family. In normal physiological regulation, EGF binds specifically to EGFR, promotes EGFR and ERK phosphorylation, and activates its downstream signaling pathway molecules to exert regulatory functions (Singh et al., 2016; Abud et al., 2021). By the results we found that the total protein level of NHE3 remained basically unchanged for a short time after activation of EGFR with EGF, while the level of surface NHE3 protein was slightly up-regulated, but prolonged EGF treatment caused a slight decrease in the protein level of NHE3. The slight upregulation of NHE3 expression on the membrane in the short term after EGF stimulation may be an acute regulation occurring within a short period of time after cell activation, and the acute regulation is rapid and reversible, so the level of surface NHE3 protein returned to normal after 60 min of EGF treatment. However, continuous EGF stimulation leads to rapid cell growth and differentiation, causing cell growth inhibition in the presence of limited space and nutrient supply, which may explain the relative decrease in NHE3 expression and mobility after continuous activation of EGFR instead. Tyrphostin AG-1478 is a selective EGFR tyrosine kinase inhibitor with clinically proven antiviral activity against HCV and encephalomyocarditis virus (EMCV) (Dorobantu et al., 2016). After using AG1478 to inhibit EGFR activity, the phosphorylation levels of EGFR and ERK were significantly down-regulated and the relative expression of NHE3 was significantly up-regulated after EGFR inhibition. The above results indicate that PEDV infection is regulated by the EGFR/ERK signaling pathway to regulate NHE3 expression, and a certain negative phase between EGFR and NHE3 (Figure 6). When EGFR activity was inhibited, the phosphorylation level of ERK was also inhibited, while the activity of NHE3 was increased. Our laboratory studies demonstrated that NHE3 activity was regulated through the EGFR/ERK pathway. Importantly, NHE3 mobility on the plasma membrane of TGEV infected cells was significantly weaker than that in normal cells, and EGFR inhibition and knockdown recovered this mobility (Yang Z. et al., 2018). Therefore, we speculate that the activity and mobility of NHE3, regulated through the EGFR/ERK pathway on the brush border membrane of small intestinal epithelial cells, decreased after PEDV infection.

In order to further study the mechanism of PEDV regulating NHE3, the expression of key signaling factors in NHE3 and EGFR pathways was detected by regulating the activity of EGFR after infection with PEDV. EGFR promotes PEDV infection to downregulate NHE3 expression and mobility (Figure 7). This result indicated that after EGFR was activated by PEDV infection, the invasion and proliferation of PEDV could be affected by EGFR, and the expression and mobility of NHE3 could be regulated. The following regulatory mechanisms exist for EGFR and NHE3 in porcine transmissible gastroenteritis virus (TGEV), which is also an alphacoronavirus: infection with TGEV enhances intestinal glucose uptake and increases the expression of EGFR, SGLT1, and GLUT2 in intestinal epithelial cells, and there is a positive regulatory relationship between EGFR and SGLT1 (Ren et al., 2013; Dai et al., 2016). In the case of TGEV infection, down-regulation of SGLT1 expression promotes the translocation of NHE3, which increases the expression of NHE3 on the plasma membrane (Yang et al., 2020). Inhibition of EGFR activity can promote the fluidity of NHE3 on the plasma membrane and promote the absorption of Na^+^ (Yang Z. et al., 2018). Combined with the results of this experiment, we preliminarily speculate that PEDV can activate EGFR, causing the decrease of NHE3 activity in small intestinal epithelial cells, and the Na^+^/H^+^ exchange barrier, which can promote the occurrence of diarrhea (Gekle et al., 2001; Dominguez et al., 2016).

**Figure 10:**
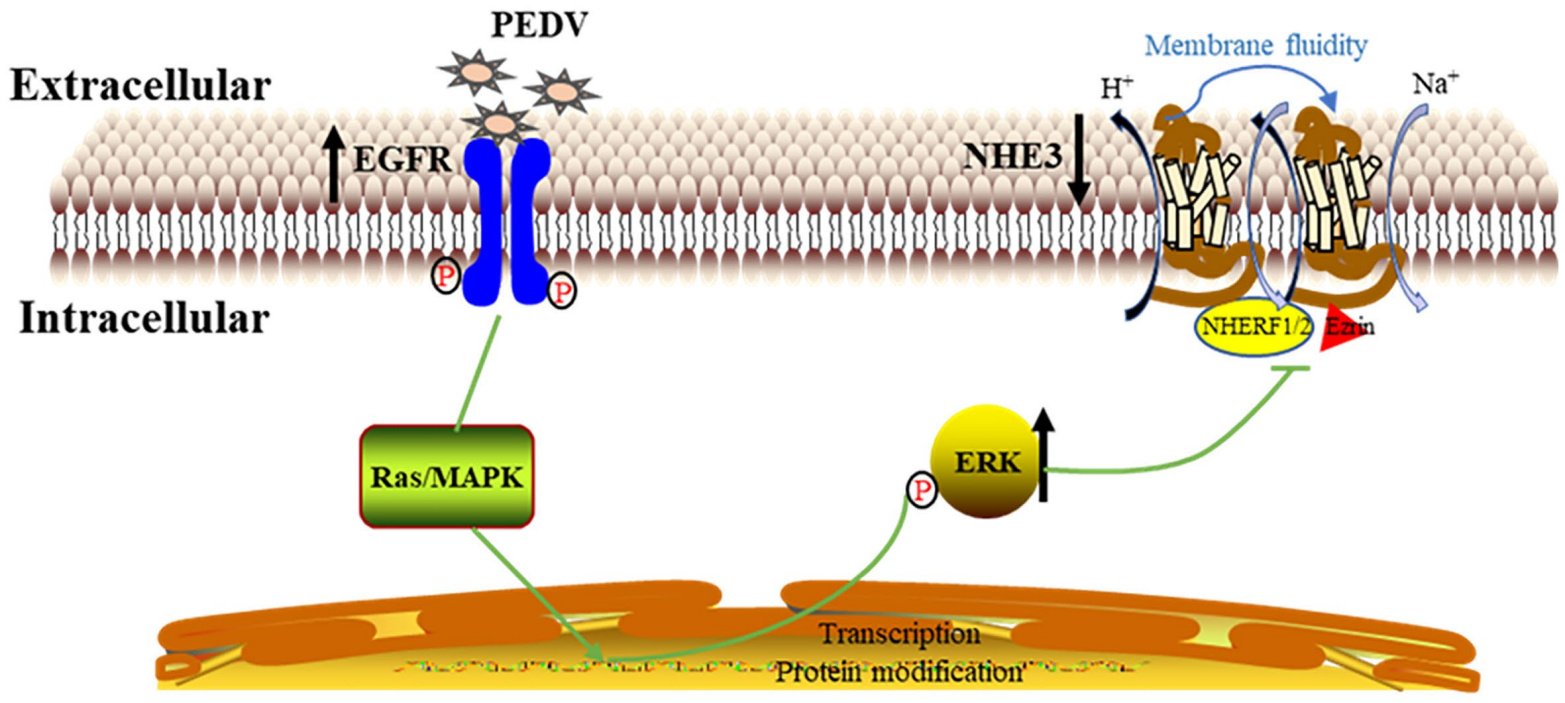
Mechanistic model of PEDV infection regulating NHE3 activity by activating EGFR.

In conclusion, EGFR may be one of the receptors involved in PEDV invasion of intestinal epithelial cells, and PEDV infection activates EGFR, which then phosphorylates ERK and thereby regulates the expression and mobility of NHE3 on the plasma membrane, ultimately leading to reduced NHE3 activity. Decreased NHE3 activity and impaired Na^+^/H^+^ exchange may be key factors in the development of diarrhea induced by PEDV infection in newborn piglets (Figure 10).

## Data availability statement

The raw data supporting the conclusions of this article will be made available by the authors, without undue reservation.

## Ethics statement

The animal study was approved by Southwest University Laboratory Animal Ethics Review Committee/Southwestern University Animal Experiment Institutional Review Committee. The study was conducted in accordance with the local legislation and institutional requirements.

## Author contributions

YZ and SZ wrote the first draft of the manuscript. ZHS, XL, and GL wrote parts of the manuscript. ZK performed the statistical analysis. HZ, SX, and JZ contributed to the conception and design of the study. ZHS organized the database. All authors contributed to the revision of the manuscript, read and approved the submitted version.
